# Time resolved 3D live-cell imaging on implants

**DOI:** 10.1371/journal.pone.0205411

**Published:** 2018-10-10

**Authors:** Alexandra Ingendoh-Tsakmakidis, Lena Nolte, Andreas Winkel, Heiko Meyer, Anastasia Koroleva, Anastasia Shpichka, Tammo Ripken, Alexander Heisterkamp, Meike Stiesch

**Affiliations:** 1 Department of Prosthetic Dentistry and Biomedical Materials Science, Hannover Medical School, Hannover, Germany; 2 Industrial and Biomedical Optics Department, Laser Zentrum Hannover e.V., Hannover, Germany; 3 Nanotechnology Department, Laser Zentrum Hannover e.V., Hannover, Germany; 4 Institute for Regenerative Medicine, Sechenov First Moscow State Medical University, Moscow, Russia; 5 Institute of Quantum Optics, Leibniz University of Hanover, Hannover, Germany; University of Vigo, SPAIN

## Abstract

It is estimated that two million new dental implants are inserted worldwide each year. Innovative implant materials are developed in order to minimize the risk of peri-implant inflammations. The broad range of material testing is conducted using standard 2D, terminal, and invasive methods. The methods that have been applied are not sufficient to monitor the whole implant surface and temporal progress. Therefore, we built a 3D peri-implant model using a cylindrical implant colonized by human gingival fibroblasts. In order to monitor the cell response over time, a non-toxic LIVE/DEAD staining was established and applied to the new 3D model. Our LIVE/DEAD staining method in combination with the time resolved 3D visualization using Scanning Laser Optical Tomography (SLOT), allowed us to monitor the cell death path along the implant in the 3D peri-implant model. The differentiation of living and dead gingival fibroblasts in response to toxicity was effectively supported by the LIVE/DEAD staining. Furthermore, it was possible to visualize the whole cell-colonized implant in 3D and up to 63 hours. This new methodology offers the opportunity to record the long-term cell response on external stress factors, along the dental implant and thus to evaluate the performance of novel materials/surfaces.

## Introduction

The use of dental implants constitutes a revolution in dentistry by restoring the tooth function in partially or fully edentulous patients. Approximately two million dental implants are placed worldwide each year [1,2]. Peri-implant inflammation might be induced by oral bacterial biofilms and leads to gradual tissue destruction and eventual implant loss [3]. According to a recent meta-analysis, the median prevalence of peri-implant infections is 26% for patients with at least 5 years implant function time and 21.2% with at least 10 years [4]. Therefore, novel antibacterial implant materials and surfaces are proposed in order to minimize the biofilm-related dental implant failure. For instance, surface coatings or laser-structured and liquid-infused surfaces have been shown to be antibacterial [5–9].

An intact biological seal, which is formed by the gingival tissue, around the implants is important for the success of implantation. The gingival soft tissue including the epithelial cells and the fibroblasts constitutes the first biological barrier against oral bacteria [10–12]. The gingival fibroblasts belong to the major gingival tissue cell types and are responsible for the normal connective tissue turnover, inflammatory response, wound healing, and regeneration [13–15]. The results from a recent study showed that oral fibroblasts are able to modulate the response of macrophages to bacterial exposure [16]. After dental implant installation, gingival fibroblasts form a collagen-rich connective tissue. This healthy tissue repopulates the wound leading to a soft-tissue seal. A good soft-tissue-implant interface, which is initiated by gingival fibroblasts, is required to form a barrier against bacterial penetration and parallel inhibition of epithelial downgrowth [9,17]. Therefore, many studies in the field of dental implant testing have been conducted using gingival fibroblasts [5–9,17–19].

The novel implant materials are typically examined for their antibacterial properties and cellular biocompatibility in 2D cultures with oral biofilms or tissue cells, respectively. Their examination has been terminal, using end-point microscopy or biochemical assays [5–9]. In order to include several time points during material testing, many material samples are required, if terminal examination methods are applied. Non-invasive imaging techniques permit to monitor the progression of events within a single sample. Non-invasive examination has been used in dentistry for the *in vivo* examination of gingival tissue applying different techniques like confocal laser scanning microscopy, cone-beam computerized imaging, and optical coherence tomography [20–22]. In addition, confocal microscopy allowed the non-invasive examination of dental surfaces [23,24]. Using those methods tissue sampling has been avoided. A non-invasive *in vitro* method to monitor biofilms on a plain surface was also successfully performed through combined nuclear magnetic resonance and confocal microscopy [25]. However, the dental implants have a cylindrical geometrical setting, which is also not reflected by the standard 2D test systems. The 2D test systems using flat samples permit a high-throughput screening as well as microscopic examination in parallel to molecular or biochemical investigation. Spatiotemporal information on how the condition of tissue cells progress and any other topographical effects along the cylindrical implant cannot be gained using these systems. Even though only surface information from the implant is needed, which can be displayed in a 2D image, the 3D geometry of the implant itself requires a 3D imaging technique to visualize the full surface. A three-dimensional non-invasive biofilm imaging technique on dental implants has been established via scanning laser optical tomography (SLOT). This technique allowed the visualization of the biofilm growth over the whole implant [26]. A similar visualization of gingival cell reactions on the implant material would complement the non-invasive and three-dimensional implant examination as well as the well-established standard 2D methods.

We aimed to introduce this new method for non-invasive monitoring of gingival fibroblast response along implant surfaces over time. We used SLOT for the visualization of cells on the implants. The SLOT offers the opportunity to image a large field of view in 3D (4 cm x 4 cm x 4 cm) on nontransparent surfaces [26,27]. For the 3D implant examination, a peri-implant model was required including human gingival cells. In order to represent the implant-cell interface, a titanium cylinder was colonized with human gingival fibroblasts. This fibroblast-colonized implant was embedded in a supporting matrix for the development of the 3D peri-implant model. It was essential to fulfill the appropriate preconditions for a compatible, stable, clear, and non-autofluorescent matrix, thus several hydrogels were tested. Furthermore, a non-toxic staining protocol for long-term LIVE/DEAD imaging suitable for SLOT was necessary. The combination of SLOT with the newly developed peri-implant model and the LIVE/DEAD staining method enabled the three-dimensional long-term visualization of cell death progression along the implant.

## Material and methods

### Tissue cell colonization of titanium implants

Primary human gingival fibroblasts (1210412, Provitro AG, Germany) were used for the three-dimensional *in vitro* peri-implant model and cultured in DMEM (FG0435, Biochrom GmbH, Germany) supplemented with 10% FBS (P30-3309, PAN-Biotech GmbH, Germany) and 1% v/v penicillin/streptomycin (A2212, Biochrom GmbH, Germany) at 37 °C in a humidified atmosphere with 5% CO_2_. In order to receive implants colonized by human gingival fibroblasts, titanium cylinders (Ø 3 mm and 3 cm length) were placed on parafilm coated 6-well plates and covered by 800 μL of a 1 x 10^6^ cells per mL human gingival fibroblasts suspension. After 4 hours at 37 °C in a humidified atmosphere with 5% CO_2_, the titanium cylinders were transferred into 15 mL tubes with filter cap (188240, Greiner Bio-One GmbH, Germany) and 1.5–3 mL culture medium. Cell culture medium was changed every 2–3 days.

### LIVE/DEAD staining for live-cell imaging

The progression of gingival fibroblast response along the titanium implant in the 3D-implant model was visualized by time-resolved LIVE/DEAD imaging. For this purpose, we established a live-cell imaging staining protocol where the human gingival fibroblasts were fluorescently labeled with the CYTO-ID Red long-term cell tracer (ENZ-51037-K025, ENZO Life Sciences Inc, USA) and any membrane-compromised cell was stained with the DRAQ7 (DR70250, Biostatus Limited, UK) DNA dye. The dyes were selected according to their emission and extinction properties, since conformance to the laser and filter specifications of the SLOT without overlapping spectra was required. Moreover, the CYTO-ID Red dye allows the cell monitoring over 10 days and the DRAQ7 dye is also stable and non-toxic. Human gingival fibroblasts were fluorescently labeled with the CYTO-ID Red according to the manufacturer’s instructions. Labeled cells were seeded on culture dishes or on titanium implants for further monitoring. The DRAQ7 dye for detection of dead or membrane-compromised cells was added directly into the cell culture medium at a final concentration of 1 μM. Gradually increasing concentrations of chlorhexidine (C9394, Sigma-Aldrich Corporation, USA) (18, 36, 72, or 181 μM) for 8 hours incubation were used to determine which concentrations allow an adequate detectability of dead gingival fibroblasts.

### Hydrogel testing

The three-dimensional peri-implant model assembly for SLOT required the use of a supporting hydrogel, thus several hydrogels were tested for their gingival fibroblast biocompatibility. The 3-D Life PVA-PEG Hydrogel with 3-D RGD Peptide (0.5 mmol/L) (09-G-001, Callendes GmbH, Germany), the Matrigel (356237, Corning, USA) and the Extracel (GS211, Glycosan BioSystems Inc, USA) hydrogel were prepared according to the manufacturer’s protocols. The Matrigel was diluted with culture medium to a final concentration of 4.7 mg/mL. The PureCol—Matrigel mixture was prepared by mixing the bovine collagen type I solution (PureCol, 5005-100ML, Advanced BioMatrix Inc, USA), Matrigel, and cell culture medium on ice to final concentrations of 0.67 mg/mL and 4.7 mg/mL, respectively. The PEGylated fibrin hydrogel was prepared as previously described [28]. The human gingival fibroblasts (5000 cells in 150 μL culture medium) were seeded on 50 μL of PEGylated fibrin hydrogels after gelation in 96-well plates and cultured for 24 or 72 hours at 37 °C in a humidified atmosphere with 5% CO_2_. For the evaluation of hydrogel cytocompatibility, Cell Proliferation Kit I (MTT) (11465007001, Roche Diagnostics GmbH, USA) and Cell Cytotoxicity Assay (LDH) (11644793001, Roche Diagnostics, Germany) were used. The Cell Proliferation assay (MTT) was performed according to the manufacturer’s protocol and all data were normalized by subtracting the blank value (wells without cells). Human gingival fibroblasts grown on tissue culture plastic served as control and were set to 100% metabolic activity. The Cytotoxicity Detection Kit (LDH) was used to measure cytotoxic effects of PEGylated fibrin hydrogel on gingival fibroblasts (experimental value) in comparison to the cells cultured on a control tissue culture plastic (spontaneous LDH release, low toxicity control). The assay was performed according to the manufacturer’s protocol. Prior measurements, a solution of 2% v/v triton-X-100 (T9284, Sigma-Aldrich Corporation, USA) in cell culture medium was added to cells to generate a high toxicity control after incubation at 37 °C in a humidified atmosphere with 5% CO_2_ for 2 hours. Cell culture medium in a 96-wells plate with or without hydrogel was used as background control. The absorbance was measured at 492 nm with a reference at 650 nm. The percentage of cytotoxicity was calculated, according to the following formula: Cytotoxicity (%) = (experimental value-low toxicity control)/(high toxicity control-low toxicity control) ×100.

### Three-dimensional peri-implant model assembly for SLOT

The three-dimensional peri-implant model consists of a colonized titanium implant with CYTO-ID Red labeled human gingival fibroblasts embedded in a PEGylated fibrin hydrogel. The fibroblast-colonized titanium cylinder was placed vertically in the middle of a flat bottom glass tube (65 x Ø 15 mm, 42779065, Karl Hecht GmbH & Co, Germany) filled with 3 mL PEGylated fibrin hydrogel (0.5 μM DRAQ7). After hydrogel gelation, DMEM without phenol red (P04-03591, PAN-Biotech GmbH, Germany, 1 μM DRAQ7) was added on top. The culture media were added on top of the 3D peri-implant model prior SLOT imaging. In the samples where cell death induction was desired, the cell culture medium was additionally supplemented with 400 μM chlorhexidine digluconate (C9394, Sigma-Aldrich Corporation, USA). A gas permeable sterile filter (391–1262, VWR International LLC, USA) was used to seal the glass tube.

### SLOT imaging

The principle of SLOT has been described in detail elsewhere [27,29]. The fluorescence signals were separated from the excitation wavelength by two bandpass filters (#46–061 + #87–752 (Edmund Optics, Germany) for CYTO-ID Red and #32–756 (Edmund Optics, Germany) + F37-694 (AHF, Germany) for DRAQ7). The CYTO-ID Red dye was imaged prior the DRAQ7 dye, which was imaged repetitively with a time lag of 7 hours. During a full 360 ° rotation 1200 projection images were acquired in total with a resolution of 1000 × 3079 pixel^2^. This resulted in a resolution of 8.5 μm/pixel and a theoretical optical resolution of 20 μm. The reconstruction was performed by the open source software IMOD [30] using a filtered back projection algorithm. Additional image processing, as calculation of the maximum intensity projections, was done in FIJI [31,32].

## Results

### LIVE/DEAD staining for live-cell imaging

**Fig 1:**
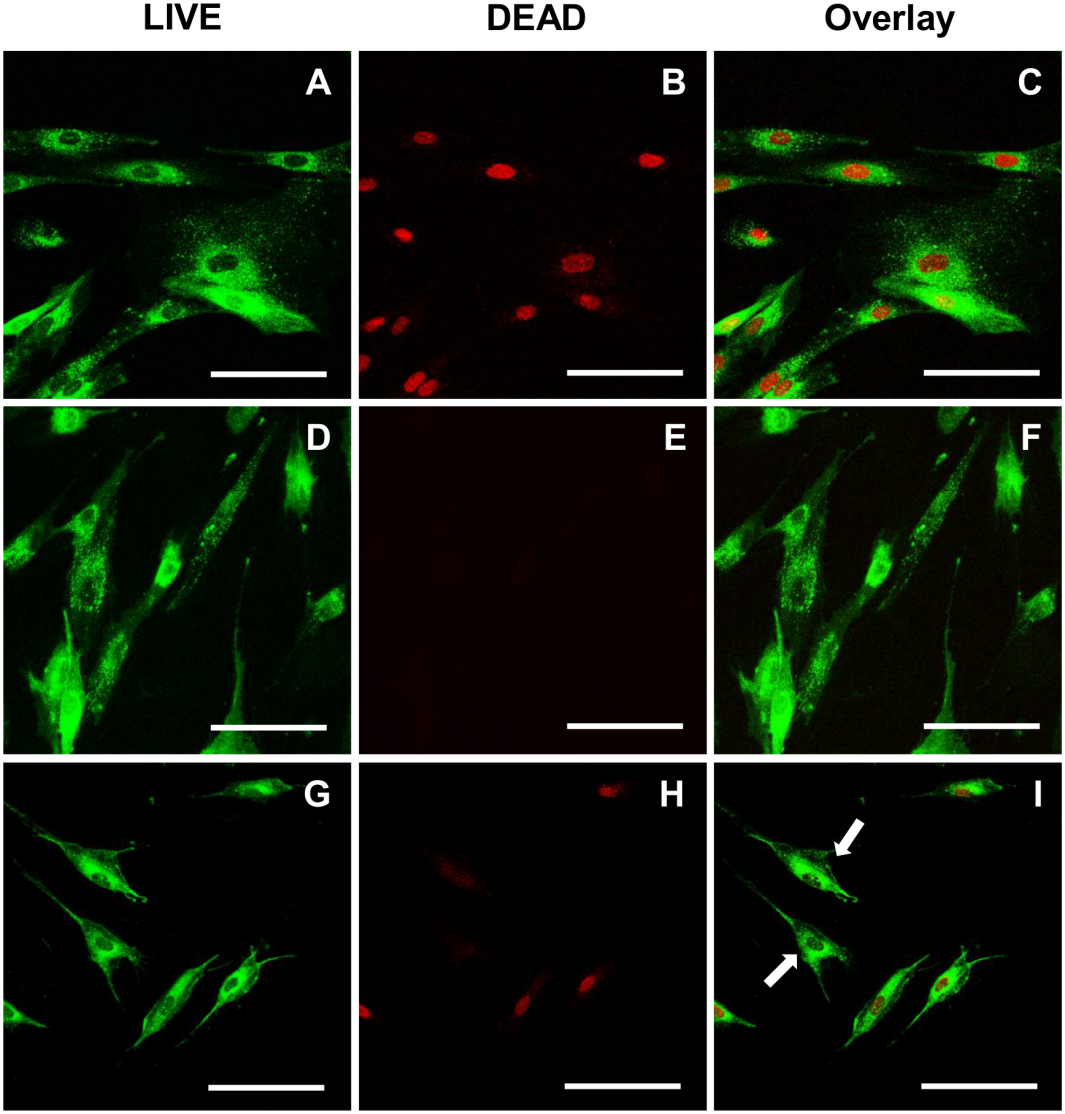
LIVE/DEAD staining of chlorhexidine treated gingival fibroblasts. CYTO-ID Red labeled human gingival fibroblasts were treated with or without chlorhexidine. CYTO-ID Red labeled gingival fibroblasts, fixed with 4% w/v PFA in PBS and subsequently stained with DRAQ7 served as a positive control for the LIVE/DEAD staining: (A) CYTO-ID Red, (B) DRAQ7, and (C) their overlay. Labeled gingival fibroblasts without addition of chlorhexidine served as a negative control after DRAQ7 staining: (D) CYTO-ID Red, (E) DRAQ7, and (F) their overlay. CYTO-ID Red labeled gingival fibroblasts were treated with 132 mM chlorhexidine for 2 hours prior DRAQ7 staining: (G) CYTO-ID Red, (H) DRAQ7, and (I) their overlay. Arrows show live cells without DRAQ7 nucleus staining. The samples were examined under the CLSM (Leica TCS SP2). Scale bars: 100 μm.

**Fig 2:**
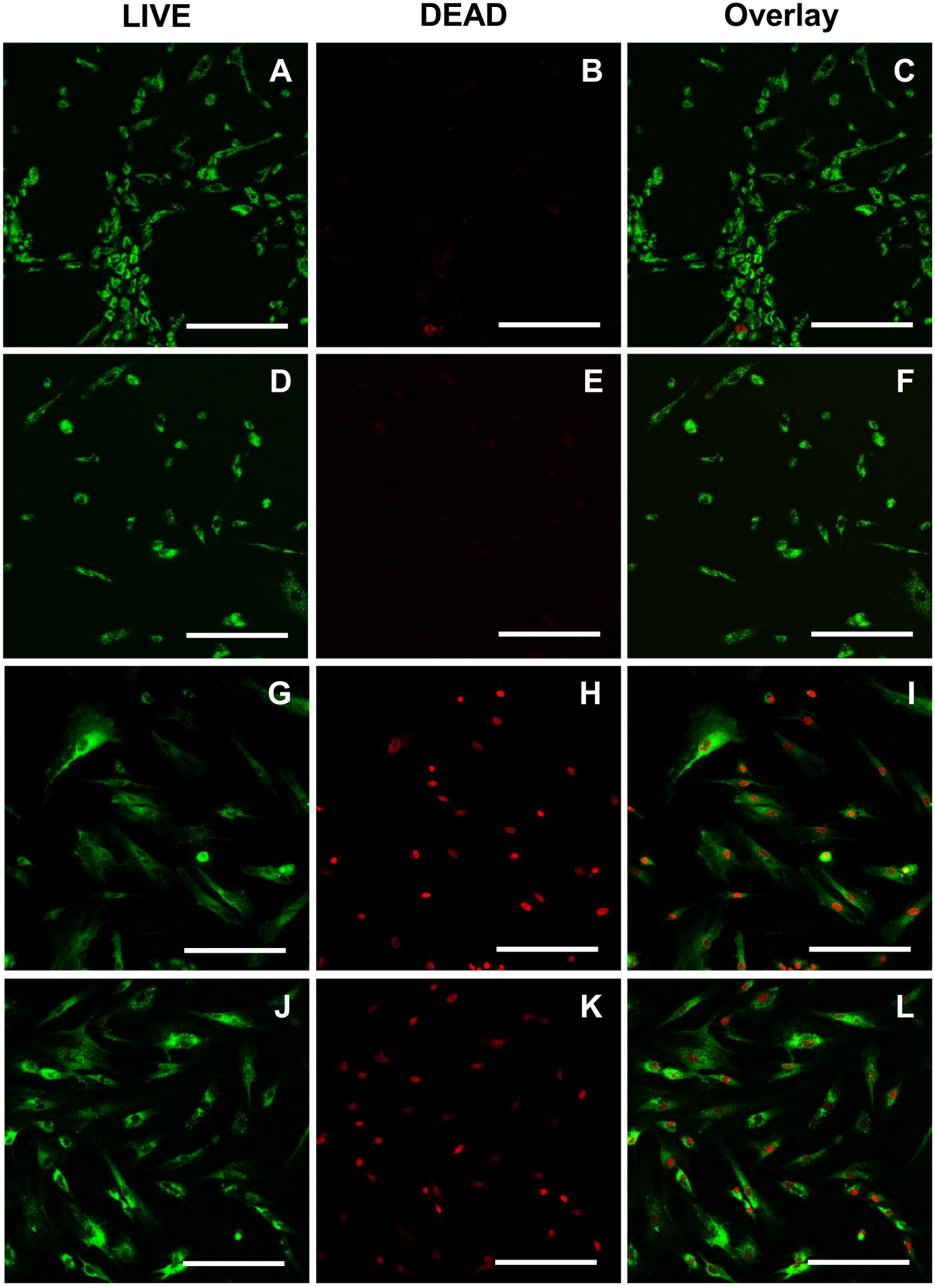
LIVE/DEAD staining of gingival fibroblasts treated with different chlorhexidine concentrations. CYTO-ID Red labeled human gingival fibroblasts were treated with different concentrations of chlorhexidine for 8 hours prior DRAQ7 staining. LIVE/DEAD stained gingival fibroblasts after treatment with 18 μM chlorhexidine: (A) CYTO-ID Red, (B) DRAQ7, and (C) their overlay. LIVE/DEAD stained gingival fibroblasts after treatment with 36 μM chlorhexidine: (D) CYTO-ID Red, (E) DRAQ7, and (F) their overlay. LIVE/DEAD stained gingival fibroblasts after treatment with 72 μM chlorhexidine: (G) CYTO-ID Red, (H) DRAQ7, and (I) their overlay. LIVE/DEAD stained gingival fibroblasts after treatment with 181 μM chlorhexidine: (J) CYTO-ID Red, (K) DRAQ7, and (L) their overlay. The samples were examined under the CLSM (Leica TCS SP2). Scale bars: 200 μm.

A LIVE/DEAD staining protocol was established which allows a 3D time resolved imaging of tissue cell response along the dental implant. The LIVE/DEAD staining distinguishes between human gingival fibroblasts with compromised cell membranes and cells with an intact cell membrane. This staining was validated in cell cultures with a 2D conformation prior its application in the 3D peri-implant model. All gingival fibroblasts on the implant were labeled with the live-cell imaging dye CYTO-ID Red. The non-toxic DRAQ7 DNA dye was able to pass cell membrane of compromised cells, since they were fixed with 4% w/v PFA in PBS (335.2, Carl Roth GmbH, Germany) to generate a positive control of cell damage Consequently, the fibroblasts had damaged cell membranes and emitted both dyes, the CYTO-ID Red was located at the cell membranes and the DRAQ7 at the nuclei (Fig 1A–1C). In contrast, no nuclei were stained in the negative control including untreated cells (Fig 1D–1F). Subsequently, we treated fibroblasts with 132 mM chlorhexidine to induce partial cell death, which confirmed the capability of the staining protocol to distinguish live from dead fibroblasts (Fig 1G–1I). Incubation of human gingival fibroblasts with chlorhexidine for 8 hours revealed that 72 and 181 μM treatments result in high percentage of fibroblasts with compromised cell membrane (Fig 2). In addition, the cell area decreased probably in response to the higher chlorhexidine concentrations and accompanied toxic effect.

### Development of a 3D tissue cell-implant model for SLOT

**Fig 3:**
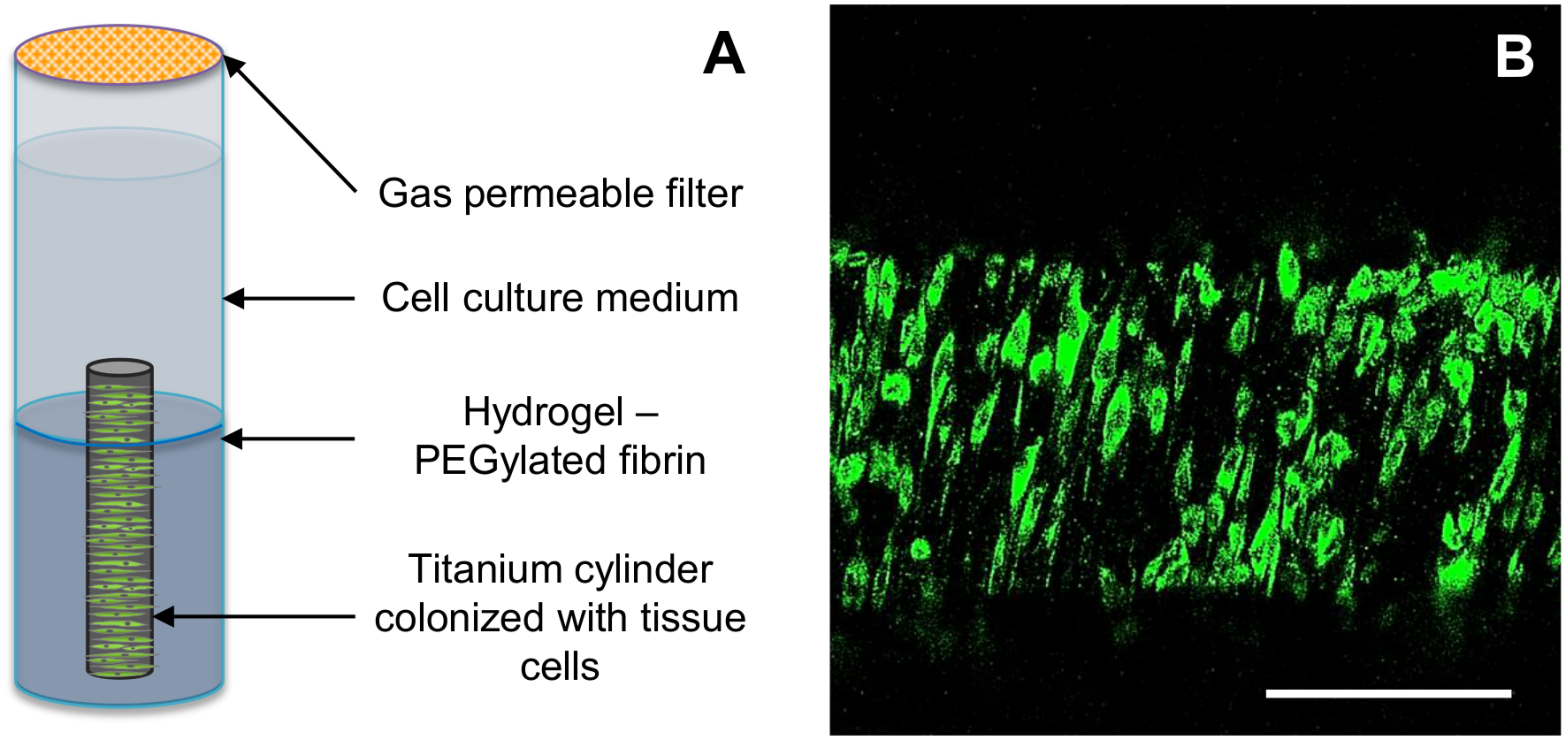
3D peri-implant model for SLOT. (A) Schematic representation of the 3D peri-implant model consisting of a colonized titanium implant by CYTO-ID Red labeled human gingival fibroblasts embedded in a PEGylated fibrin hydrogel. The fibroblast-colonized titanium cylinder was placed vertically in the middle of a flat bottom glass tube filled with the hydrogel. A gas permeable sterile filter was used to seal the glass tube. (B) Titanium implant colonized by CYTO-ID Red labeled human gingival fibroblasts (green). Scale bar: 200 μm.

**Fig 4:**
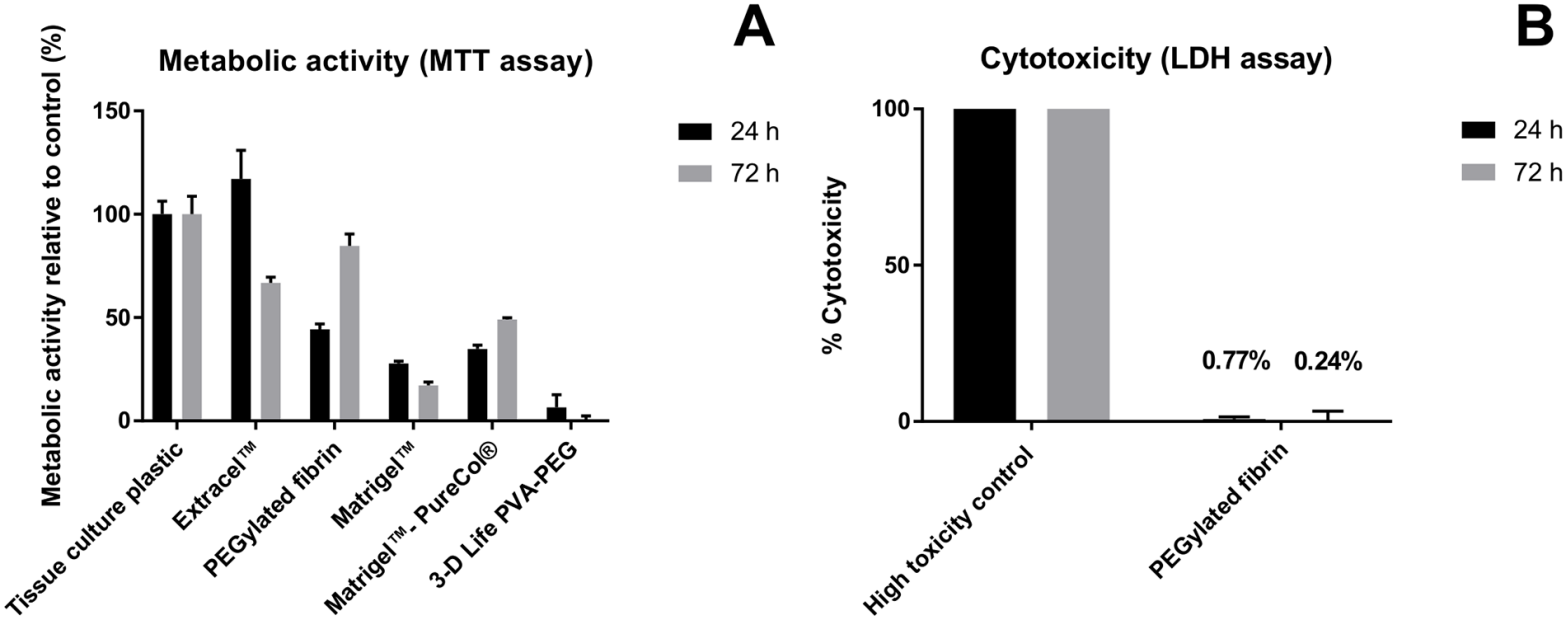
Cytocompatibility of tested hydrogels. (A) Metabolic activity of gingival fibroblasts grown on various hydrogels for 24 and 72 hours measured by the Cell Proliferation Kit I (MTT). The metabolic activity is shown as percentage relative to control, tissue culture plastic, which was set to 100%. (B) Cytotoxicity of the PEGylated fibrin hydrogel on gingival fibroblasts after 24 and 72 hours measured by the Cell Cytotoxicity Assay (LDH). The percentage of cytotoxicity was calculated in relation to the low (spontaneous LDH release from fibroblasts grown on tissue culture plastic) and high (fibroblasts grown on tissue culture plastic treated with 2% v/v triton-X-100) toxicity controls.

The SLOT 3D imaging technique imposes additional demands on the 3D model. This comprises the optical accessibility of the dental surface and the elimination of fluorescent background. The model consists of a fibroblast-colonized implant embedded in a supportive hydrogel in a glass tube (Fig 3A). The titanium implants were successfully colonized by CYTO-ID Red labeled human gingival fibroblasts (Fig 3B). Furthermore, a biocompatible, non-fluorescent, and stable hydrogel was required. Several hydrogels were tested for their cytocompatibility. The PEGylated hydrogel displayed the closest metabolic activity to the control after 72 hours (Fig 4A). The fibroblasts on the other hydrogels reached lower metabolic activities. After 24 hours, Extracel showed a high metabolic activity, which declined after 72 hours. Hence, Extracel was excluded. In addition, Matrigel and PureCol exhibited high autofluorescence which would disturb the final measurements. The cytocompatibility of the PEGylated fibrin gel was confirmed by LDH cytotoxicity assay with fibroblasts (Fig 4B). Consequently, we used the PEGylated fibrin hydrogel for the assembly of the 3D peri-model.

### SLOT measurements

**Fig 5:**
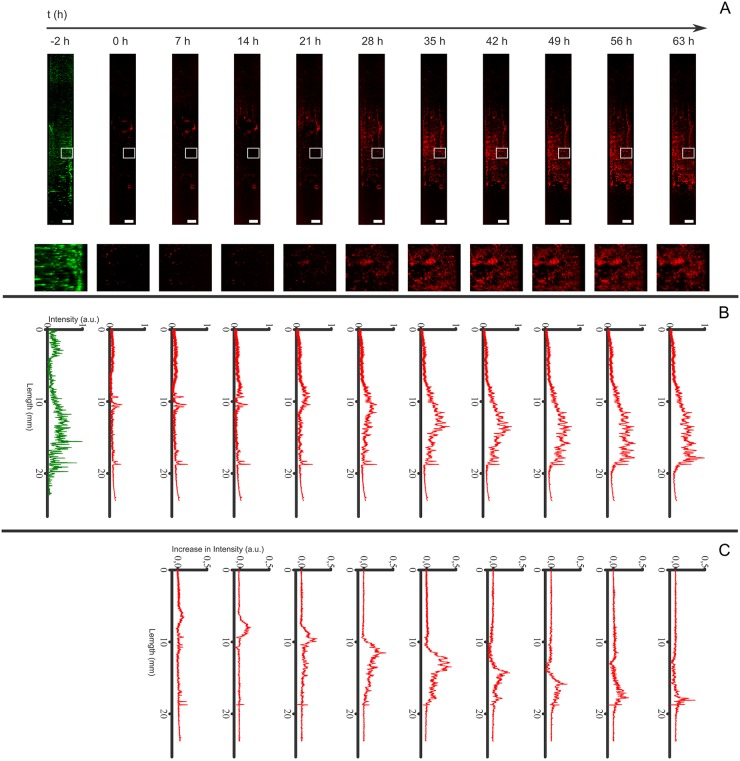
Maximum intensity projections (MIP) for the individual time points and corresponding fluorescence intensity profile along the titanium implant after the addition of chlorhexidine. (A) MIP of the live cell stain with CYTO-ID Red (green) and dead cell stain with DRAQ7 (red) at the different time points. Rectangles indicate area of zoomed in versions of each MIP. (B) Fluorescence intensity profile of the MIPs (see corresponding image above in A). The profile was measured top-down and averaged for the full width of the titanium implant. (C) The difference spectrum for consecutive profiles in B.

**Fig 6:**
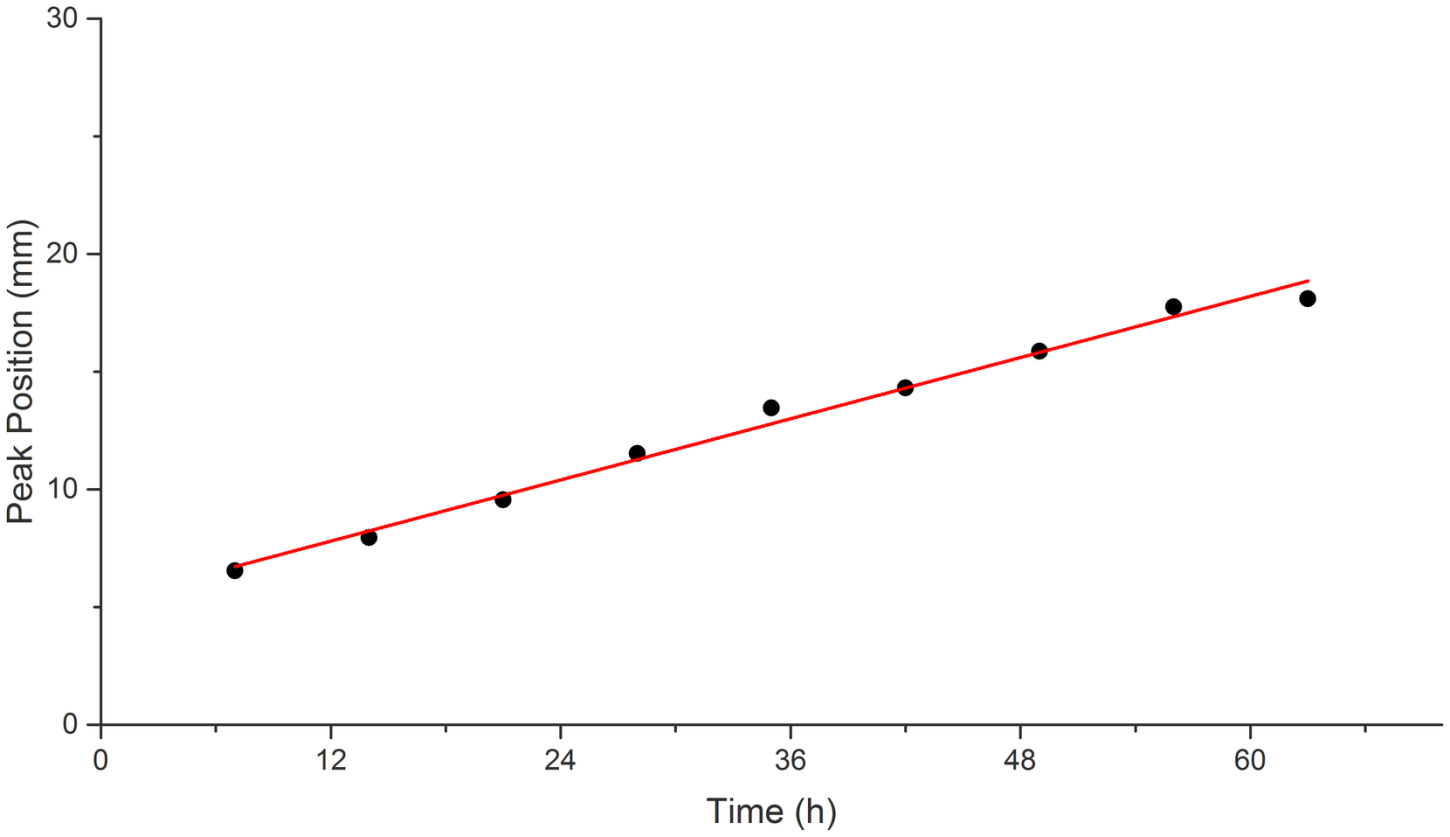
The intensity increase location on the titanium implant after chlorhexidine treatment plotted versus the time. The location of the peak in Fig 5C was determined and plotted versus the time (black dots). A linear fit was performed (red line), resulting in a slope of 0.2 mm/h and a coefficient of determination of R^2^ = 0.988.

The 3D peri-implant models were measured using SLOT every 7 hours for 63 hours. The spatiotemporal progression of cell death along the implant was monitored after the addition of toxic chlorhexidine concentration (400 μM) on top of the peri-implant model (Fig 5). The maximum intensity projections (MIP) of the reconstructed datasets are shown in Fig 5A. The first MIP shows the CYTO-ID Red stained (green) gingival fibroblasts, which were distributed over the whole titanium implant. The first measurement of the DRAQ7 stain, which depicts dead gingival fibroblasts, was set as time point 0 hours. At this time point, only few dead cells could be observed on the titanium implant. With increasing time, the intensity of the dead cell stain increased and moved downward, along the implant. This was also visualized in a 3D rendering (see Supporting Information S1 Movie). For quantification, the intensity profiles of individual measurements were generated from top to the bottom of the cylinder and averaged for the full width of the titanium implant (see Fig 5B). The maximum value was normalized to 1 for both, the live and dead cell stain. The increase in intensity and the movement to the bottom were reconfirmed. Furthermore, the spectrum for the dead cell stain for later time points approximates the intensity distribution of the live cell stain. By calculating the difference spectrum for two succeeding profiles of the dead cell stain, the new gained intensity for each time point was determined. These profiles showed a peak that moved over the time from top to bottom (see Fig 5C). The peak was always accompanied by a proceeded drop in intensity. The location of the maximum of each spectrum in Fig 5C was determined and plotted in Fig 6 against the time. A linear fit was performed, which resulted in a slope of 0.2 mm/h with a coefficient of determination of R^2^ = 0.988. The same measurements were performed on a control sample without the addition of chlorhexidine (see Supporting Information S1 and S2 Figs). The cell death increases over time also in the control sample, however, homogenously distributed over the full length of the titanium implant.

## Discussion

An intact implant-mucosa interface is necessary to avoid oral biofilm growth along the implant into the tissue [10–12]. The reaction of soft-tissue cells from up to down along the implant is of great interest, since external stress progression, like biofilm growth, is apical [33]. In order to complement the high-throughput 2D testing with spatiotemporal information, a non-invasive and time resolved imaging in a three-dimensional setting of the cell reaction would be of advantage. We established a three-dimensional peri-implant model and a LIVE/DEAD staining, which allowed the non-invasive monitoring of cell death progression along the implant over time.

The establishment of a LIVE/DEAD staining for live-cell imaging allowed the monitoring of cell death progression up to 63 hours, without toxic adverse reactions. After toxic chlorhexidine treatment, it was possible to distinguish live from dead human gingival fibroblasts (Fig 1). In addition, high chlorhexidine concentrations led to a high number of dead cells (Fig 2), indicating that a high concentration is required in the 3D model to achieve a cell toxic effect. We tested several hydrogels for the model development and could determine the most suitable in terms of stability, cytocompatibility, and autofluorescence (Fig 4). Fibrin is a naturally derived blood clotting protein, which can be prepared in a hydrogel form. It has been previously shown that fibrin promotes cell attachment and growth [34,35]. However, fibrin gels undergo degradation and strong matrix remodeling *in vitro*, which results in construct shrinkage and weight loss. PEGylation of fibrinogen is one possible method to control the gel stability without impairment of its excellent cytocompatible properties [28]. In this study PEGylated fibrin was evaluated highly cytocompatible with gingival fibroblasts. In addition, in comparison to other gels, PEGylated fibrin remained fully transparent upon gelation, which represents an optical benefit and enables deep microscopic analysis of cells cultured within this hydrogel. Using the PEGylated fibrin as a supportive material it was possible to detect the stained fibroblasts on the titanium implants via SLOT technique.

The effect of toxic chlorhexidine treatment on gingival fibroblasts in the established three-dimensional peri-implant model was successfully demonstrated by non-invasive time resolved monitoring of cell death path along the implant. Fig 5 shows the progression of chlorhexidine associated cell death path from the top to the bottom of the peri-implant model. The linear fit in Fig 6 shows the velocity of the chlorhexidine diffusion. The diffusion of the chlorhexidine molecules resulted in a constant velocity along the implant surface. The intensity drop in Fig 5C is most probable due to photo bleaching, which is possible only if the DRAQ7 passed the membrane-compromised cell and is bound to the DNA. The DRAQ7 fluorophore in the higher part of the implant is exited repetitively and suffers more from photo bleaching, than the DRAQ7 in the lower part of the implant that was only exited once. The cell death on the control sample, which increases after 49 hours, might be due to lack of constant nutrient supply and culture conditions (S1 Fig). In order to perform continuous acquisition, the samples were at standard room conditions and no medium exchange was done. In the control sample, the intensity gain location related to cell death is not linear indicating a lack in cell death pattern (S2 Fig). In contrast, a linear cell death progression was determined in the chlorhexidine treated sample (Fig 6). This observation confirms that the cell death progression along the implant is caused by chlorhexidine, which diffused from top to bottom through the hydrogel.

To conclude, we could develop a model for non-invasive LIVE/DEAD monitoring of cell response along implant surfaces over time in combination with SLOT. To the best of our knowledge, this is the first report of non-invasive and spatiotemporal monitoring of cell condition along implant surface. Due to its complexity this 3D LIVE/DEAD monitoring is not applicable for high-throughput analysis. Moreover, it is currently not possible to collect molecular or biochemical data. However, this method will help to monitor non-invasively the cell response on the whole dental implant and by that to complement the typical 2D *in vitro* investigations of implant materials. Questions on how tissue cells react to biofilm downgrowth along the implant can be addressed by our proposed method. Such investigations apply to basic research as well as to translational if patient tissue cells or oral biofilms and real dental implants are used.

## Conclusions

We developed a method for non-invasive optical monitoring of live cell response in an *in vitro* 3D peri-implant model. To the best of our knowledge, this is the first demonstration of non-invasive imaging of dynamic change of the cell condition on an implant in response to external stress. The proof of principle, demonstrated in this work, allows the development of optically based imaging tools for the analysis of more sophisticated *in vitro* 3D cell-implant models (e.g. co-cultured with oral bacterial biofilms). Thus, this represents a unique experimental platform for tissue engineering and implant material testing in combination with oral microbiology.

## Supporting information

S1 Fig Maximum intensity projections (MIP) for the individual time points and corresponding fluorescence intensity profile along the titanium implant without the addition of chlorhexidine. (A) MIP of the live cell stain with CYTO-ID Red (green) and dead cell stain with DRAQ7 (red) at the different time points. Rectangles indicate area of zoomed in versions of each MIP. (B) Fluorescence intensity profile of the MIPs (see corresponding image above in A). The profile was measured top-down and averaged for the full width of the titanium implant. (C) The difference spectrum for consecutive profiles in B.
(TIF)

S2 Fig The intensity gain location on the titanium implant without chlorhexidine treatment plotted versus the time. The location of the peak in S1C Fig was determined and plotted versus the time (black dots). No systematic behavior of the intensity gain can be determined. The peaks seem to appear randomly over time.
(TIF)

S1 Movie 3D rendering of the cell death progression along the implant. The DRAQ7 intensity distribution is shown in 3D for all time points from 0h—63 hours in steps of 7 hours. 3D rendering was performed using Voreen (voreen.uni-muenster.de).
(MP4)
