# Supplementary figures and images for: Time resolved 3D live-cell imaging on implants
Source: PLoS One. 2018 Oct 10;13(10):e0205411. doi: 10.1371/journal.pone.0205411 (PMC6179276; doi:10.1371/journal.pone.0205411)

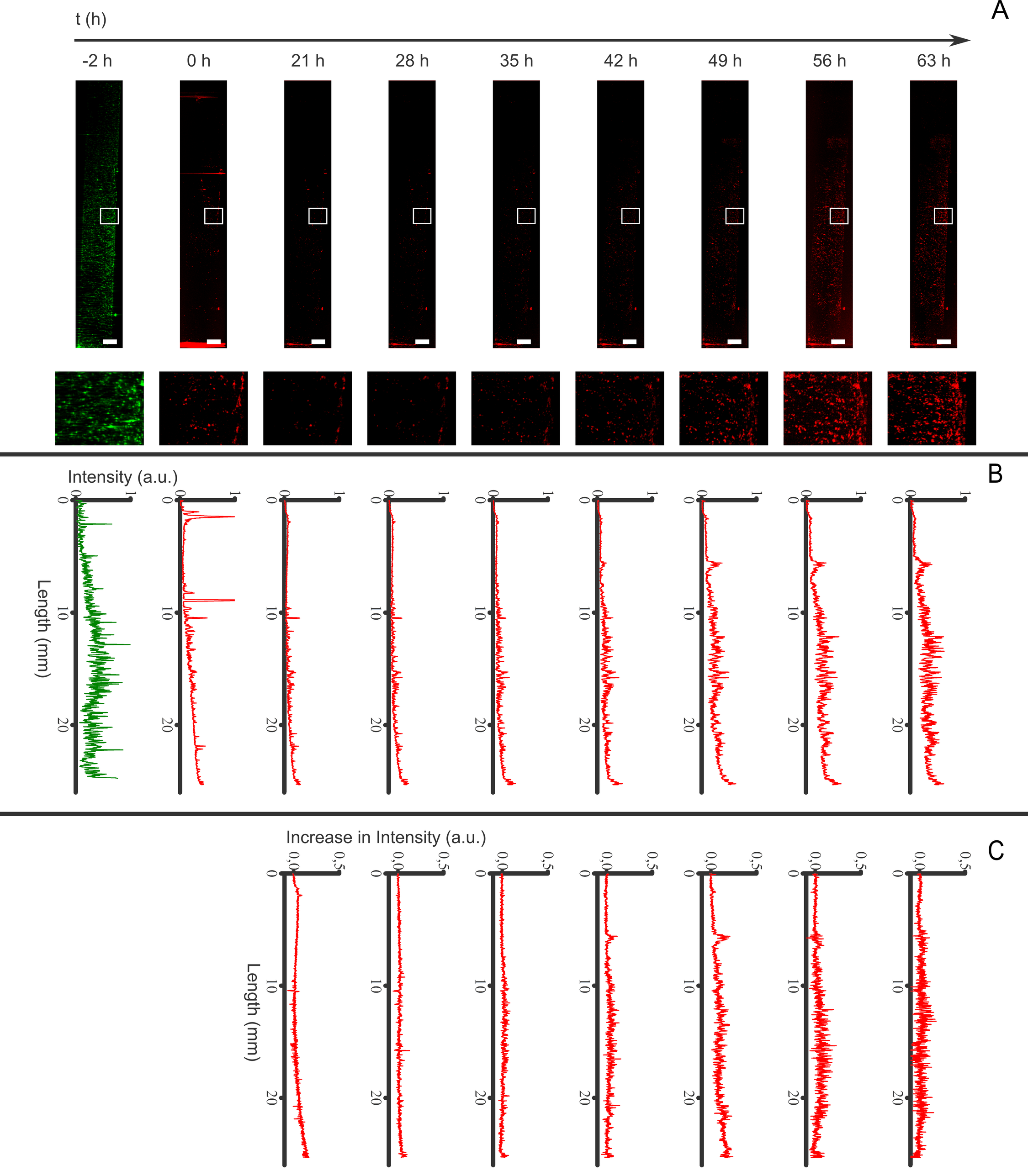

Supplement: S1 Fig — (A) MIP of the live cell stain with CYTO-ID Red (green) and dead cell stain with DRAQ7 (red) at the different time points. Rectangles indicate area of zoomed in versions of each MIP. (B) Fluorescence intensity profile of the MIPs (see corresponding image above in A). The profile was measured top-down and averaged for the full width of the titanium implant. (C) The difference spectrum for consecutive profiles in B. (TIF) [file pone.0205411.s001.tif]
